# Eliciting Distributive Preferences in Health Care Resource Allocation: A Person Trade-Off Study

**DOI:** 10.3390/healthcare13111309

**Published:** 2025-05-30

**Authors:** Nan Fang, Chang Su, Jing Wu

**Affiliations:** 1 School of Pharmaceutical Science and Technology, Faculty of Medicine, Tianjin University, Tianjin 300072, China; kldfangn@tju.edu.cn (N.F.); 2022213073@tju.edu.cn (C.S.); 2 Centre for Social Science Survey and Data, Tianjin University, Tianjin 300072, China

**Keywords:** health care priority setting, resource allocation, distributive justice, person trade-off, threshold effects

## Abstract

Background/Objectives: While a preference for an equal distribution of health gains is common, there are situations where individuals may opt to concentrate health gains for a select few. This study investigates how distributive preferences, defined as societal valuations of alternative allocations of fixed total health benefits, vary with the magnitude of individual health gains. Methods: Using the person trade-off (PTO) method, we conducted an online survey with a nationally representative sample of Chinese adults (N = 500). The respondents evaluated five allocation programs differing in both individual health gain magnitude and number of beneficiaries. Distributive preferences are classified into five distinct types: diffusion, concentration, maximization, extreme egalitarianism and extreme inequality seeking. Threshold regression analysis identified critical transition points in preference patterns. Results: Non-maximizing tendencies were dominant (79% of the respondents). The health gain threshold was estimated to be 4.6 years (95% CI: [4.28, 4.85]): below this threshold, respondents tend to allocate smaller benefits to more patients (diffusion preference); above the threshold, people are inclined to allocate larger benefits to fewer patients (concentration preference). The income level and self-reported health status of the participants were identified as potential factors influencing distributive preferences. Conclusions: This study provides the first quantitative evidence from China that distributive preferences exhibit a non-linear shift based on the magnitude of health benefits. The identified 4.6-year threshold provides policymakers with an empirically based instrument to strike a balance between efficiency and the reduction in inequality in resource allocation. These findings advocate for incorporating social value weights into health technology assessments, especially for interventions that offer substantial individual benefits.

## 1. Introduction

### 1.1. Background and Significance

The allocation of scarce health care resources remains a critical challenge for health systems globally. Cost-effectiveness analysis (CEA) has been widely used to inform the priority setting for health care resources around the world. Quality-adjusted life years (QALYs), a metric of health gain combining both quality and length of life, is the dominant measure of health outcomes for use in CEA. However, there are many concerns about the appropriateness of using QALYs to inform scarce health care resource-allocation decisions [1]. One of the criticisms is that the QALYs are based on the measurement of individual utility, yet the values elicited are used to inform social choice [2]. It is argued that the QALY approach overlooks equity and equality, as each additional QALY is implicitly considered to have the same social value regardless of who obtains it and how the total QALY gains are distributed across patients, e.g., large gains to the few are equally valuable as small gains to the many [3,4].A systematic review collected critiques of QALY-based decision making, emphasizing its failure to address equity concerns, particularly for vulnerable populations [5]. The use of QALYs potentially determines the health care system’s goal of pursuing efficiency, which is to maximize the sum of individual QALYs. This limitation is exacerbated in contexts like China, where socioeconomic disparities and cultural values may reshape public preferences for health benefit distribution. Yet policymakers lack empirical guidance on how citizens weigh efficiency against distributive justice. This study addresses this gap by investigating the distributive preferences to inform the resource-allocation decisions in China.

### 1.2. Literature Review

#### 1.2.1. Theoretical Foundations of Distributive Preferences

Several methods have been suggested for incorporating efficiency and equality trade-offs in a consistent way [6,7,8]. Most of them use the social welfare function (SWF) in health, borrowing from modern welfare economics, which is also called the health-related social welfare function (HRSWF). The use of isoelastic SWF in health was put forward to allow some trade-offs between the maximization of the sum of health and its distribution [8]. This isoelastic SWF has its origins in a concept proposed to address income distribution inequality [9]. One of the advantages of the isoelastic SWF is that the strength of social preferences for equality can be summarized in a single parameter, the inequality aversion parameter [8].

However, being able to capture the trade-off between efficiency and equality in a single parameter comes at the cost of reduced flexibility [10]. The implicit assumption in the HRSWF is that a wider spread of health gains is consistently preferred to a greater concentration when people distribute health gains. In this case, the indifference curve of social welfare will always be convex to the origin, exhibiting a diminishing marginal rate of substitution [8]. However, some empirical investigations have revealed that people’s distributive preferences do not always align with the predictions of the HRSWF [11,12,13]. Individuals sometimes exhibit a preference for concentrating rather than diffusing health gains.

#### 1.2.2. Empirical Evidence

Empirical research on distributive preferences in health care resource allocation, mainly conducted in Western countries, has explored the factors influencing these preferences, consistently revealing threshold-driven preference shifts. It has been reported that 55.8% of respondents preferred concentration among senior officials in Canada, challenging the assumption of equal distributional preferences [11]. Similar research in the Norwegian general population showed that when the health gain is very small, there may be a preference for substantial improvements for the few rather than insignificant improvements for the many. This research also suggested that there might be a minimum threshold value that is required before the gains are considered worth diffusing, which lies somewhere around 1 year (assessed 1, 2 and 5 years) [12]. Subsequently, a pilot study involving 61 undergraduate students in Spain reported a minimum threshold of 9.1 years (assessed 1, 2, 5, 20 and 50 years) [13]. This indicates that preferences shift from concentration to diffusion as health gains exceed the threshold. Other studies have also reported that the minimum threshold is approximately 2.6 years in the United Kingdom (UK) or 6 months in Denmark [14,15]. These differences highlight the need for context-specific research, as most studies have been Western-based, leaving Asian preferences unexamined.

#### 1.2.3. Methodological Advances and Challenges

The person trade-off (PTO) method is the primary approach used in the empirical research mentioned above to estimate the social value of different health care interventions. The core principle of PTO involves presenting participants with a trade-off between the number of beneficiaries and the magnitude of health gains. Specifically, it aims to determine how many individuals receiving intervention A are equivalent to X individuals receiving intervention B, thereby enabling a comparison of their relative social values.

PTO, which was first developed in 1973 [16], has been widely adopted in recent studies. For example, age-weighted preferences were incorporated into PTO tasks to enhance policy relevance [17]. This method was also applied to compare the relative social value of the non-health benefits from pediatric gene sequencing versus health benefits from adult treatments [18]. By simulating societal decision-making scenarios, PTO positions respondents as decision makers. However, this approach may impose cognitive burdens due to its complex trade-off nature [19].

Furthermore, PTO results are susceptible to framing effects, including the methodology eliciting equivalence values (e.g., direct questioning vs. iterative adjustment) [20], whether a ‘no preference’ option is permitted [21], and the order in which scenarios are presented (‘order effect’) [22]. In studies of distributive preferences, the order effect refers to the fact that the sequence of presenting health benefit magnitudes in PTO tasks may lead to response bias.

#### 1.2.4. Dynamic Contexts and Policy Implications

Emerging technologies and environmental stressors are redefining global health priorities. In 2023, the UK’s National Institute for Health and Care Excellence (NICE) endorsed weighting schemes for ‘highly specialised technologies’, recognizing that substantial health gains justify preferential resource allocation—marking a policy shift driven by threshold considerations [23]. In China, such adaptations must consider localized equity concerns, as highlighted in analysis of low- and middle-income countries [24].

#### 1.2.5. Research Gaps

Although health gain distribution has been widely studied [3,5,25,26], several gaps remain. First, the existing literature has a limited focus on the Asian population. Asian countries have very different sociocultural and economic contexts from those of Canada and European countries, where most of this work has been conducted [11,12,13,14,15]. It has been found that only 5% of health priority-setting studies focused on Asia, with none addressing China’s urban–rural divide [25]. Initial investigations also revealed the urban–rural inequality of opportunity in health care in China [27]. Second, considering the dynamic nature of society, with rapid technological advancements, environmental changes [28], and economic development, there is a need to update the research on distributive preferences, as people’s values and preferences may have evolved over time. Finally, the impact of respondents’ characteristics on distributive preferences is unclear.

### 1.3. Research Objectives and Contribution

Thus, the aims of the paper are as follows. First, it aims to investigate whether the public’s distributive preferences for concentration of individual health gains still exist in the Asian population and the current socioeconomic context. China’s unique sociocultural and economic environment, with rapid urbanization and a mixed public–private health system, offers an ideal setting to test these preferences. Second, the study seeks to quantify the health gain threshold at which distributive preferences shift from preferring diffusion to concentration.

This paper contributes to the literature by, firstly, being the first study to explore the pattern of change in distributive preferences for concentration or diffusion in the Chinese population, which has important implications for health care resource-allocation decision making; secondly, it provides different distributive preferences for groups of people with different characteristics; and finally, it examines the influence of the starting point of the size of individual health benefits on the distributive preference methodologically. For instance, the health benefit increases from 1 year to 50 years, compared with a decrease from 50 years to 1 year.

## 2. Materials and Methods

### 2.1. Person Trade-Off Methodology

The PTO technique was employed to quantify the social value of different sizes of individual health gains and subsequently infer people’s distributive preferences (concentration or diffusion) [29,30]. In this approach, respondents are presented with a trade-off between two different programs, which can benefit different numbers of people. Through repeated PTO tasks relative to a common comparator, the relative social value among different programs can be determined [29,31]. Given the aim of this study, a set of programs with different sizes of health gains were evaluated, yielding a quantitative estimate of the relative social value of large health gains for a few versus small health gains for many.

### 2.2. Survey Design

The respondents were asked to imagine that they were making decisions about how to allocate the available health care budget, choosing between two competing health care programs (labeled ‘A’ and ‘B’) that can improve the health of different patient groups, differing in both the size of the health gains per patient and the number of beneficiaries. The size of the health gains of programs A and B and the number of patients in program A are provided to the respondents. They were then asked to determine the number of patients to be treated under program B so that there was no difference between the two programs. That is, the total social values of program A and program B are the same [12]:

(1) $$u\left(P,t\right)=u\left(p,T\right),$$

where *u* refers to the social value of health care programs; *P* and *p* refer to the number of patients who received gains in programs A and B, respectively; and *t* and *T* refer to the health gains that each patient received in programs A and B, respectively. By specifying the values for *t*, *P* and *T* using the PTO technique, the value of *p* can be derived to achieve indifference between the two programs.

Both programs targeted 20-year-old patients to avoid any potential effect of patient age on decision making. Apart from the specified differences, no other variations existed between the health care programs. Health gains are measured in life years in full health to make them easier to understand, which has also been successfully utilized in prior empirical studies exploring distributive preferences [12,13,15]. In addition, research suggests that respondents in different pilot surveys find it more meaningful to compare different health gains in terms of improvements in quantity of life [32].

Five PTO tasks were designed (Table 1). In each task, the health gains and the number of beneficiaries in program A differed. To assess the validity of the distributive preference change model proposed in previous studies [12,13] within our sample, this study employed the same set of health benefit magnitudes used in prior research, enabling direct result comparisons. Specifically, *t* = 1, 2, 5, 20 and 50 years in program A, and *T* = 10 years in program B. To isolate the impact of individual health gain magnitude on distributive preferences, while controlling for total health gain variations, the total number of life years gained in program A (*P* × *t*) was set to be the same in all five PTO tasks (*P* × *t* = 100 years).

### 2.3. Person Trade-Off Tasks

**Figure 1:**
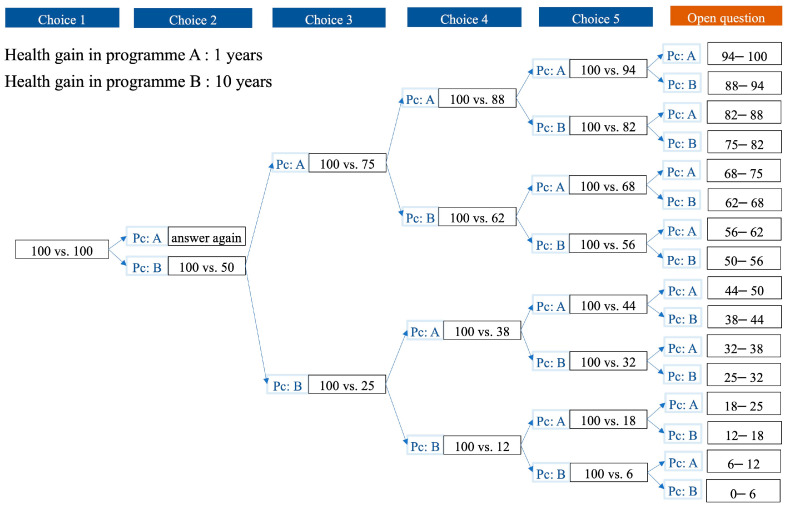
Iterative process in person trade-off tasks (using *t* = 1 year as an example). Pc: previous choice. The black box shows the number of people who will benefit from program A vs. program B. The iterative progress to the other four PTO tasks can be found in Supplementary Material S1.

Two versions of the questionnaires were developed to control for ‘starting point bias’. In version 1, respondents completed the five PTO tasks in order of *t* from least to most, while in version 2, they completed the tasks in descending order. Given the limitations of the cognitive overload inherent in the PTO approach, it was difficult for respondents to answer the indifference number of patients directly. Therefore, we used a three-step approach to help respondents approach the indifference point through a series of successive questions (see Figure 1, taking *t* = 1 year as an example).

The first step was a choice task. We presented respondents with a pairwise choice (choice 1) between programs that would benefit the same number of people. For example, program A includes 100 people with whom everyone can live an additional year in full health; program B includes 100 people with whom everyone can live an additional 10 years in full health. The respondents were asked ‘which of these two programs would you choose to fund?’ A rational respondent would surely choose the program with greater health gains. In cases of an unexpected choice, the questioning was paused, allowing respondents to reconsider their answer or exit the questionnaire.

The second step was based on the bisection approach [33]. If the size of the health gains in program A was smaller than that in program B (i.e., *t* = 1, 2 and 5 years), a follow-up choice that halves the number of people of the program preferred in the previous choice would be presented, and the less preferred program would be left unchanged. Subsequent choices continued this process of bisecting the gap between the upper and lower limits (based on previous choices) to iterate towards an interval of indifference between the two programs (as shown in Figure 1). If the size of the health gains in program A was greater than that in program B (i.e., *t* = 20 and 50 years), a follow-up choice with 100 people for the less preferred program in the previous choice was presented, and the more preferred program was left unchanged. For example,

**Choice** **1.** A: 5 people each gain 20 years vs. B: 5 people each gain 10 years (choose A)

**Choice** **2.** A: 5 people each gain 20 years vs. B: 100 people each gain 10 years

If the respondent chose B in choice 2, the following choices would use a bisection approach to reduce the number of people in program B to iterate towards an interval of indifference between the two programs. For example,

**Choice** **3.** A: 5 people each gain 20 years vs. B: 50 people each gain 10 years

If respondents still choose A in choice 2, we would directly ask the respondents, ‘How many people who can be treated by program B within the range of 100–200 would make you consider the two programs no different?’ [34]. See Supplementary Material S1 for the iterative process of other sizes of health gains.

The third step involved an open-ended question. Once the indifference interval for the number of beneficiaries between the two programs was narrowed to approximately 5 people, to obtain a more precise indifference point, respondents were asked ‘How many people in this range would think the two programs were the same?’.

### 2.4. Study Sample and Data Collection

The PTO tasks were administered via an online survey. A total of 500 participants were recruited through ‘Kurun’ (an existing commercial internet panel). The inclusion criteria for participants were Chinese citizens aged ≥18 years and currently residing in mainland China. Exclusion criteria included those who refused to participate in the survey and were unable to read the text. To ensure representativeness of the general Chinese population, participants were selected based on age, gender, education level, urban and rural areas, and geography. The specific quota was determined based on data from China’s seventh population census (see Supplementary Material S2). Each respondent was rewarded with credits, which could be exchanged for cash when they reached a certain amount as financial compensation for participation. The survey was conducted between March and May 2022. Following a face-to-face pilot survey (Supplementary Material S3), the first round of the formal survey was carried out, and two weeks later, a second round was administered to approximately half of the first-round respondents to assess choice path repeatability.

Section 1 of the survey collected sociodemographic information about the respondents, including age, sex, education level, marital status, employment status, income, area of residence, medical insurance and self-reported general health. Section 2 presented the five PTO tasks. Two orders of the tasks were randomly assigned to all the respondents. Section 3 asked for feedback about the difficulty of the questionnaire. See Supplementary Material S4 for an overview of the questionnaire for PTO tasks.

### 2.5. Statistical Analysis

The statistical analysis consisted of three primary and three secondary analyses. In the primary analyses, we first assessed the respondents’ distributive preference for concentration or diffusion based on the equivalence value (p) and calculated the proportion of different distributive preferences in each PTO task. Next, we further conducted two hypothesis tests to test whether the distributive preference exists and, if so, whether it changes with the size of the health gain. Finally, if the distributive preferences do change, the threshold at which distributive preferences change was obtained by fitting the SWF. Secondary analyses included testing for ‘starting point bias’, testing for test–retest reliability, and subgroup analysis.

#### 2.5.1. Distributive Preference Type Analysis

We first performed a descriptive analysis of the indifference number of patients in program B (p) across all 500 responses for each PTO task. The range of *p* allowed us to infer the distributive preferences of the respondents and to classify them into five categories [12]. The specific classification for each distributive preference type is shown in Table 2. Those categorized as ‘maximizing’ represent that people are only concerned with the overall health gain. Those categorized as ‘diffusion’ represent people who prefer small gains for many people to large gains for few people, those categorized as ‘concentration’ represent people who prefer large gains for few people to small gains for many people. Those categorized as ‘extreme egalitarianism’ represent that people prefer a large number of beneficiaries, regardless of how small the health benefit is. Those categorized as ‘extreme inequality seeking’ represent that people strongly prefer large health gains, regardless of how small the number of beneficiaries is.

It is also possible that p reached the upper or lower limit of the available interval simply because respondents refused to make a trade-off between the number of beneficiaries and the size of the benefits. Unfortunately, we were unable to differentiate between them and those who had extreme preferences but were still willing to trade off, as the final open-ended question was a drop-down box option that everyone had to answer. Therefore, to avoid confounding, these respondents were uniformly classified as nontrade-offs in the subsequent analysis. The percentages of respondents for each preference type in the five PTO tasks will be calculated and compared to gain a preliminary understanding of the distributive preferences for the concentration or diffusion of individual health gains and to determine their changes as the size of health gains increases.

#### 2.5.2. Testing the Hypotheses

We calculated the mean value of p for each PTO task ($\bar{p}$), which allowed us to conduct formal statistical tests of the following two hypotheses about distributive preferences from the mean level. To exclude the effect of extreme values on the calculation of the mean, as long as there is an extreme preference in either PTO task, the respondent was excluded.

**Hypothesis** **1.** *Distributive preferences for the concentration or diffusion of individual health gains do not exist in our population. Each additional QALY has the same social value.*

We tested Hypothesis 1 by using the Student’s *t*-test to check whether $\bar{p}$ was not significantly different from ten.

**Hypothesis** **2.** *As the size of individual health gains changes, people’s distributive preferences shift between diffusion and concentration.*

This hypothesis is accepted if the existence of preferences for both concentrating and diffusing depends on the size of the health benefits. We define preferences for concentration and diffusion as follows. Suppose there are two programs to be compared and their total health benefits (number of beneficiaries × individual health gains) are equal: $P_{1}\times t_{1}=P_{2}\times t_{2}$. In addition, by conducting PTO tasks, we also know that $u\left(P_{1},t_{1}\right)=u\left({\bar{p}}_{1},10\right)$ and $u\left(P_{2},t_{2}\right)=u\left({\bar{p}}_{2},10\right)$, where ${\bar{p}}_{1}$ and ${\bar{p}}_{2}$ refer to the number of patients each receiving 10 years, which can be as good as program 1 and program 2, respectively. Therefore, the size of ${\bar{p}}_{1}$ and ${\bar{p}}_{2}$ can be used as a substitute for the social value of program 1 and program 2. If $P_{1}$ > $P_{2}$ and ${\bar{p}}_{1}$ < ${\bar{p}}_{2}$, the respondents perceived that the program with fewer beneficiaries to be of greater value and were defined as preferring to concentrate health gains. Conversely, if $P_{1}$ > $P_{2}$ and ${\bar{p}}_{1}$ > ${\bar{p}}_{2}$, respondents were defined as preferring to diffuse health gains. To compare as many distributive preferences as possible in relation to different sizes of health benefits, the Student’s t-test was used to compare the differences in the $\bar{p}$ values of any two of the six programs (*t* = 1, 2, 5, 10, 20 and 50 years), resulting in a total of 15 pairs of comparisons, as shown in Table 3.

#### 2.5.3. Estimating the Threshold

To quantify the distributive preference threshold, we use the SWF proposed by Rodriguez-Miguez and Pinto-Prades (2002) [13], in which positive inequality aversion and negative inequality aversion can be allowed to coexist:

(2) $$u\left(t\right)=\alpha_{1}e^{-\alpha_{2}t}t^{\alpha_{3}},$$

where *t* indicates the number of QALYs received from the program, $u\left(t\right)$ is the social value of t, and $\alpha_{1}$ and $\alpha_{2}$ are the parameters. To obtain the estimated $\alpha_{3}$, we linearized Equation (2) by means of a log transformation, obtaining Equation (3):

(3) $$lnu\left(t\right)=ln\alpha_{1}-\alpha_{2}t+\alpha_{3}lnt$$

If we assume a constant social value of increasing numbers of persons [32], Equation (1) changes to (4):

(4) $$P\times u\left(t\right)=p\times u\left(T\right)$$

Given that the utility function can be scaled arbitrarily without changing its properties, and that *T* was always 10 years in our PTO tasks, we specified *u* (10) = 10. Hence, the social value of the health gains *t* is expressed as:

(5) $$u\left(t\right)=10\times \frac{p}{P}$$

Respondents who did not make trade-offs between the number of persons and health benefits were excluded from the SWF analysis, because if the subjects do not make trade-offs, their preferences cannot be represented by a continuous SWF [13]. We estimated the function using ordinary least squares regression, with adjusted (degrees of freedom) R^2^ as a measure of goodness of fit. The regression was used as a method to identify ‘tipping points’ where preferences shift. From the shape of the estimated u(t), it can be inferred that if people prefer to diffuse (inequality aversion), u(t) should marginally decrease because the social value per unit of health gain decreases as the size of the benefit increases, and vice versa. Then, we can find the health gain threshold at which preference changes through the shape of u(t). We adopted 1000 bootstrap replications to calculate the percentile confidence interval (CI 95%) of the threshold.

#### 2.5.4. Consistency Analysis and the Test–Retest Reliability

The $\bar{p}$ in each PTO task was calculated in the two versions of the questionnaire with different orders of the PTO task, and the Student’s *t*-test was used to check whether the mean results of two versions of the questionnaire were significantly different. The test–retest reliability of PTO responses was assessed by using the intraclass correlation coefficient (ICC) [35]. Test–retest reliability is a statistical method for evaluating the consistency of measurement results of measurement tools or methods at different time points. ICC (Intraclass Correlation Coefficient) is a commonly used indicator for evaluating the test–retest reliability. It is usually used to assess the stability of the measurement results of the same group of individuals at different time points or when measured by different evaluators. The mean ICCs of all the respondents were calculated.

#### 2.5.5. Subgroup Analysis

To explore the influence of population characteristics on the response results, we conducted subgroup analysis of different populations on the basis of the characteristics collected, including income level, age, education level and self-reported health status. The average indifference number of people in program B ($\bar{p}$) and health gain thresholds of each subgroup were calculated. The $\bar{p}$ values in different subgroups were compared with the total population using the Student’s *t*-test, and Bonferroni correction was performed.

## 3. Results

In total, 500 respondents from 177 cities in 28 provincial-level administrative regions in China completed the PTO questionnaire. These participants, all meeting the quota-required demographic characteristics, were representative of the Chinese population. The participants had an average age of 44.7 years, with 49.8% being female and 24.8% having an educational level of primary school or lower. The sample covered both low-income and high-income groups. Additionally, 36.2% of participants resided in rural areas. Supplementary Material S5 summarizes the respondents’ demographic characteristics. The average time to complete the questionnaire was 20 min (10–40 min). The second-round survey was completed by 242 respondents (follow-up rate is 48.4%).

### 3.1. Distributive Preference Type

The indifference numbers of patients in program B (p) across all 500 responses for each PTO task are presented in Supplementary Material S6 (Figures S5–S9). These figures also demonstrate that respondents were not answering the survey questions at random; otherwise, an equal proportion of respondents would have answered for each possible *p*. Table 4 shows the distribution of different types of distributive preferences inferred from the range of *p* values answered by the respondents. It is easy to find that preferences for concentrating and diffusing both exist in our sample, and people’s preferences may change with the size of health benefit. The proportions of concentration as compared with diffusion were 0.08 (7.2%/89%) when *t* = 1, 0.39 (24.0%/63.2%) when *t* = 2, 1.08 (37.6%/33.6%) when *t* = 5, 19.05 (78.0%/4.0%) when *t* = 20, and 4.41 (74.6%/17.4%) when *t* = 50. When *t* = 1, 2 and 5, there was significantly less concentration and significantly more diffusion than when *t* = 20 or 50.

After excluding 68 respondents with extreme preferences, among the remaining 432 respondents, the mean indifference number of people in program B in the five PTO tasks ($\bar{p}$) is shown in Table 5. If people only intend to maximize the QALY gains, $\bar{p}$ should equal 10. However, when *t* = 1, 2, 20 and 50 years, $\bar{p}$ is significantly greater than 10; when *t* = 5, $\bar{p}$ is significantly less than 10. Therefore, Hypothesis 1 is rejected at the 5% level. Thus, ‘each additional QALY has the same social value’ is not consistent with the preferences for resource distribution in the Chinese population. In addition, there was no statistically significant difference between the results of the two versions of the questionnaires in any of the five PTO tasks.

Table 3 shows the results for Hypothesis 2. We find that as *t* increases, people’s distributive preferences begin to shift from diffusion to concentration. People’s distributive preferences are statistically significant for all comparison pairs except for *t* = 1 year vs. *t* = 50 years. Therefore, Hypothesis 2 is accepted at the 5% level.

### 3.2. Regression Analysis Results and Threshold

The results of the regression analysis of the individual-level observations to obtain the parameters of Equation (3) are shown in Table 6.

Rewriting Equation (3) in its original form, we obtain the utility function:

(6) $$\hat{u(t)}=2.085e^{0.035t}t^{0.646}$$

**Figure 2:**
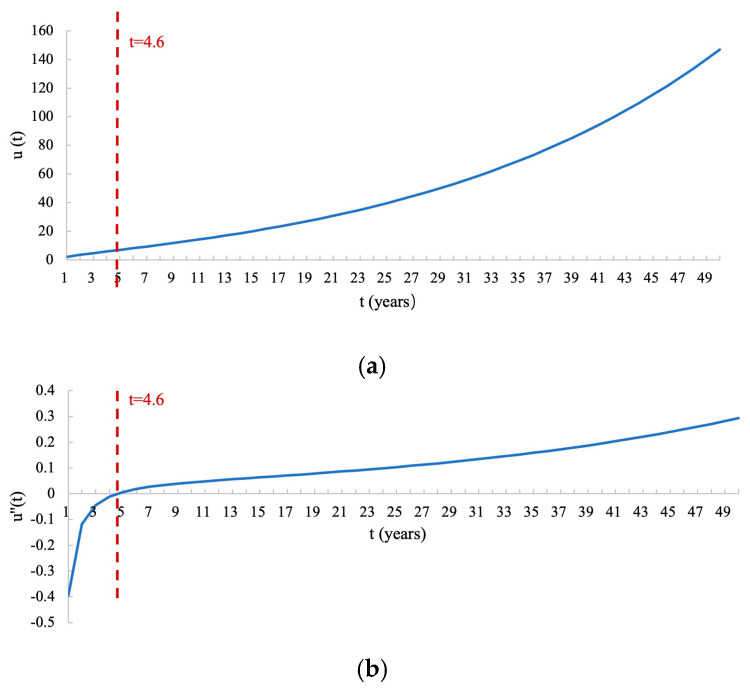
Utility function curves. (**a**) Curve of u(t); (**b**) curve of $u^{\prime \prime }(t)$. $u^{\prime \prime }(t)$ is negative for *t* values under 4.6 years and positive for *t* values greater than 4.6 years, indicating that u(t) is a marginally decreasing and convex function before *t* = 4.6 years and is a marginally increasing and concave function after *t* = 4.6 years. *t*: life years in full health that each patient received; $u\left(t\right)$: the social value of t.

The curve of the u(t) function is depicted in Figure 2a. Figure 2b shows the curve of the second derivative of u(t), denoted as $u^{\prime \prime }(t)$. As seen, u(t) is a marginally decreasing and convex function before *t* = 4.6 years (95% CI: [4.28, 4.85]) and is a marginally increasing and concave function after *t* = 4.6 years. This gives the size of the threshold of health gains. When the health benefits are less than 4.6 years, respondents tend to prefer giving small benefits to more people. However, when health benefits exceed 4.6 years, respondents tend to shift towards providing greater benefits to fewer people.

### 3.3. Test–Retest Analysis and Subgroup Analysis Results

The test–retest reliability results for five PTO tasks (Supplementary Material S7) suggest a high stability of preferences with respect to small health gains but a weaker stability with regard to large health gains. Most of the respondents could repeat their choice entirely between the two rounds of surveys on the basis of the results of individual-level data analysis.

Subgroup analysis revealed that income level, age, education level, and self-reported health status may be important factors influencing people’s distributive preferences. For people with personal monthly income between CNY 5000 and CNY 10,000 (approximately USD 690–USD 1380), the $\bar{p}$ estimates were significantly smaller than those of the total population, except for when *t* = 20 and *t* = 50. As a result, it is more difficult for people with higher incomes to change their preference from spreading out to concentration. The threshold for people in this subgroup was 4.9 years, which is larger than the 4.6 years for the total population. For people with self-reported general health status, the $\bar{p}$ estimates were significantly smaller than those of the total population, except for when *t* = 20 and *t* = 50, indicating the relatively weaker preferences for greater health benefits of other health status people. Interestingly, this phenomenon was not common in people with different ages. The distributive preferences of the elderly (≥60 years old) for allocating substantial health benefits aligned with those of younger adults aged 18 to 40. The detailed results of the subgroup analysis can be found in Supplementary Material S8.

## 4. Discussion

In this study, we examined whether there is a change in the pattern of distributive preferences and quantified the threshold for a change in the size of health gains. Our main findings are that most respondents were not health-benefit maximizers and that the degree of health gain was an important attribute influencing social value. We found that respondents’ distributive preferences may change from diffusion to concentration, and the respondents generally preferred to provide smaller benefits to more people when the health benefits were less than 4.6 years in full health. However, when health benefits exceed 4.6 years, respondents tended to shift towards providing greater benefits to fewer people. Also, we found that the starting point of the size of health benefits shown to respondents had no effect on the results.

These results confirmed the findings of previous studies that found that preferences for the diffusion and concentration of health gains both exist when social health care resources are distributed and change depending on the size of health gains [12,13,15]. Notably, in addition to the previous study’s finding of distributive preferences for concentration when health gains are very small, our study also revealed distributive preferences for concentration when health gains are very large (Table 3 and Table 4, when the size of health gains is 20 years and 50 years). As reported in previous research, when health gains are too small to be perceived as resulting in meaningful improvements in health, people prefer to make substantial improvements to the few [12]. Our study also revealed that when health gains are sufficiently large, people also prefer to provide greater health benefits to fewer people. Perhaps they consider that a large benefit can enable the recipient to have a meaningful quality or length of life, which also confirms, to some extent, the results of two other exploratory studies [36,37]. Regarding the distributive preferences for concentration in the two cases above, one possible explanation might be related to the size of the relative differences in individual health benefits between the two programs being compared [12]. Generally, when the relative differences are small, equity concerns may dominate. The differences are considered too small to justify any discrimination between the groups [12]. Conversely, we suspect that when the relative differences become large enough, discrimination between the groups begins to emerge. There are two methods to increase the differences in benefits between two programs: One is to reduce the size of the benefit of one program to be sufficiently small relative to the larger gain, so that people feel it is too small to diffuse. This was also what previous studies have utilized, and the ‘minimum threshold’ was estimated [15]. Another method is to increase the size of the benefit of the other program to be sufficiently large relative to the smaller gain, so that people think that concentrating the health gains on fewer people can produce greater social value. This is what our study did, and we estimated the ‘maximum threshold’. However, this is only a preliminary conjecture. Future research is warranted to explore the potential effects of these two thresholds, taking into account the broader size of health benefit.

We also found some framework issues related to the application of the PTO method. Firstly, the indifference number of people in program B is partly related to the upper limit of the range of people provided for respondents to answer (see Table 4). For example, when *t* = 1 it is 100, when *t* = 2 it is 50, and when *t* = 5 it is 20. Notably, when *t* = 20 and 50, the upper limit of the range provided to respondents to answer was 200, which was much higher than 100, but the indifference number was smaller than the value when *t* = 1. However, this does not affect the results of the change pattern of distributive preference, because the indifference number of people in program B is compared with 10 people to determine whether respondents prefer concentration or diffusion, which has nothing to do with the absolute value of the indifference number. Secondly, the indifference number of people in program B created some clustering which corresponds to the values in the drop-down box in the open-ended question. From the Figures S5–S9 in Supplementary Material S6, we can see that respondents were more likely to choose the larger number of beneficiaries in each drop-down box. This suggests a response pattern specific to bounded open-ended questions in PTO tasks, where respondents tended to respond to treating more patients when asked to specify the number of treated patients. Future research could consider replacing the drop-down boxes of open-ended questions with fill-in-the-blank questions to mitigate this potential bias.

This study provides empirical evidence that the significant increase in social value caused by high health benefits should be considered. For example, assigning additional weight to significant QALY gains, as the National Institute for Health and Care Excellence (NICE) has been recommended in its economic evaluation guidelines for highly specialized technologies [23]. The 4.6-year threshold challenges the traditional QALY maximization paradigm. Policymakers could use this value to prioritize therapies offering transformative benefits (e.g., cancer immunotherapies) while maintaining equality for smaller, widespread gains (e.g., hypertension management).

It should be noted that the results of this study need to be distinguished from broader research on health inequality, which mainly explored how to allocate health resources among individuals differing in social, economic, demographic, geographical or other aspects (e.g., gender, race, disability) [24]. On the one hand, these studies are more about exploring the influence of the characteristics of beneficiaries on the value of health benefits, while this study explores the influence of the characteristics of health benefits themselves (such as the magnitude of the benefits) on the value of the benefits. On the other hand, these studies assumed that people are always averse to inequality. But our study found that when the individual health benefits are very large, people also prefer to concentrate the large health benefits on a few beneficiaries, that is, to seek inequality. It has been pointed out that we need to go beyond the concept of an ‘inequality aversion parameter’.

Finally, this study has several limitations that should be acknowledged. First, this study included only six sizes of health benefits. There were no shorter-than-1-year benefits to detect the existence of the minimum difference threshold, and the 20- and 50-year health benefits appeared to be somewhat too large, limiting the accuracy of the utility curve *u*(*t*) and the derivation of the maximum difference threshold. Future studies on the health benefits of more subtle changes are needed, such as 8, 10, or 15 years, but the cognitive burden on respondents should also be taken into account. Second, the results of PTO responses may be sensitive to the decision context, for example, the age of the patients. In this study, only 20-year-old patients were modeled to avoid a possible age effect and to be able to apply a larger health benefit. However, given the lower LE of older individuals, the social value of increased health gains in the last years of life, even if they are large, may be lower, so the results may underestimate the maximum difference threshold for older patients. In addition, patients in other age groups can be modeled to extend the analysis. Third, the test–retest reliability results suggest lower reliability in responses to the large-gain scenarios (*t* = 20 and 50), with ICCs below 0.5. This indicates the instability of distributive preferences when greater health benefits are gained, and this should be considered when interpreting the results. Fourth, we did not include the ‘equally good’ options in the PTO choice, which may introduce systematic biases due to the forced-choice nature of the task. However, we posit that this omission does not affect the estimation of the threshold. To illustrate, consider a respondent who believes 100 people each gaining 1 year of health equals 25 people each gaining 10 years. In our task, when forced to choose, there is a 50% chance they will be directed to an open-ended question for a value between 18 and 25 (max fill-in 24), and a 50% chance for a value between 25 and 32 (min fill-in 26). The expected answer is 25 (25 = 24 × 50% + 26 × 50%). Including the ‘equally good’ options might also reduce the data quality. Choosing the ‘equally good’ option could indicate that respondents opted out of making a trade-off, they did not understand the task, they interpreted it as a ‘don’t know’ substitute, or they just rushed through the survey [19]. It has been warned that researchers should be cautious to interpret ‘equally good’-options as reflecting well-considered indifference values [12]. Finally, the use of the internet panel may introduce relevant potential selection biases: (1) the sample is mainly composed of people who frequently use the internet or are used to using smartphones; (2) people with limited text reading and comprehension skills are essentially excluded from the survey.

## 5. Conclusions

To address the trade-off between the maximization of the sum of health and its distribution, the standard HRSWF approach satisfies the requirements in most cases and assumes that people are always averse to inequality. However, there are also special cases. When the individual health benefits are very large, people also prefer to concentrate the large health benefits on a few beneficiaries, i.e., to seek inequality. This empirical study of the general public in China found that when the health benefits were small, there was a general tendency to provide smaller benefits to a larger number of beneficiaries. However, when the health benefits became large (above a certain threshold), people tended to provide larger benefits to fewer beneficiaries. Decision makers should consider carefully whether they are underestimating the value of interventions that provide large benefits.

## Figures and Tables

**Table 1:** Size of health gains and the number of beneficiaries in five PTO tasks.

Program A		Program B	
*t*	*P*	*T*	*p*
1 year	100 patients	10 years	?
2 years	50 patients	10 years	?
5 years	20 patients	10 years	?
20 years	5 patients	10 years	?
50 years	2 patients	10 years	?

Note: *t*: the health gains that each patient received in program A; *P*: the number of patients who received the gains in program A; *T*: the health gains that each patient received in program B; *p*: the number of patients who received the gains in program B whose respondents were indifferent between the two programs.

**Table 2:** The definitions of the five categories of distributive preferences.

Preference Category	Preference Definition
Concentration ^1^	If *t* < *T*, *p* < *Pt*/*T* If *t* > *T*, *p* > *Pt*/*T*
Diffusion ^2^	If *t* < *T*, *p* > *Pt*/*T* If *t* > *T*, *p* < *Pt*/*T*
Maximizing ^3^	*p* = *Pt*/*T*
Extreme egalitarianism ^4^	If *t* < *T*, *p* = upper limit If *t* > *T*, *p* = lower limit
Extreme inequality seeking ^4^	If *t* < *T*, *p* = lower limit If *t* > *T*, *p* = upper limit

Note: *t:* the health gains that each patient received in program A; *T*: the health gains that each patient received in program B. *T* = 10 years in every PTO task; *p:* the number of patients who received the gains in program B that make respondents indifferent between programs A and B; *P*: the number of people benefiting from program A. ^1^ When *t* < *T* (*t* = 1, 2, and 5 years, *T* = 10 years), program B represents a concentrative distribution program. If the respondent believes that the number of people benefiting from program B need not be large, even fewer than 10 people, to be as good as program A is, this indicates that the respondent prefers program B and that they have a distribution preference of concentration. When *t* > *T* (*t* = 20 and 50 years, *T* = 10 years), program A represents a concentrative distribution program. If the respondent believes that the number of people benefiting from program B should be more than 10 people to be as good as program A is, this indicates that the respondent prefers program A and that they have a distribution preference of concentration. ^2^ The range of *p* for ‘Diffusion’ was opposite to that for ‘Concentration’. ^3^ If the respondent is only concerned with the overall health gain (‘Maximizing’), *p* should always be 10, resulting in 100 person-years of benefit. ^4^ The *p* values for ‘extreme egalitarianism’ and ‘extreme inequality seeking’ reached the upper or lower limit of the range available to answer the open-ended questions, depending on which program has the smaller individual benefit.

**Table 3:** Changes in distributive preferences across different sizes of health gains.

Pairs	Program 1			Program 2			$t_{2}-t_{1}$	Distributive Preference	*t*-Test (*p* Value)
	$t_{1}$	$P_{1}$	${\bar{p}}_{1}$	$t_{2}$	$P_{2}$	${\bar{p}}_{2}$			
1	1	100	38.0	2	50	17.4	−1	D *	<0.001
2	1	100	38.0	5	20	9.4	−4	D *	<0.001
3	1	100	38.0	10	10	10.0	−9	D *	<0.001
4	1	100	38.0	20	5	32.2	−19	D *	<0.001
5	1	100	38.0	50	2	37.7	−49	D	0.42
6	2	50	17.4	5	20	9.4	−3	D *	<0.001
7	2	50	17.4	10	10	10.0	−8	D *	<0.001
8	2	50	17.4	20	5	32.2	−18	C *	<0.001
9	2	50	17.4	50	2	37.7	−48	C *	<0.001
10	5	20	9.4	10	10	10.0	−5	C *	<0.001
11	5	20	9.4	20	5	32.2	−15	C *	<0.001
12	5	20	9.4	50	2	37.7	−45	C *	<0.001
13	10	10	10	20	5	32.2	−10	C *	<0.001
14	10	10	10	50	2	37.7	−40	C *	<0.001
15	20	5	32.2	50	2	37.7	−30	C *	<0.001

Note: D: preferences for diffusing; C: preferences for concentrating; $t_{1}$ and $t_{2}$: size of health gains in program 1 and program 2 (years in full health); $P_{1}$ and $P_{2}$: number of beneficiaries in program 1 and program 2; ${\bar{p}}_{1}$ and ${\bar{p}}_{2}$: indifference number of beneficiaries in program B (health gains = 10 years) when comparing program 1 with program B and program 2 with program B. * Significant at the 5% level; $H_{0}$: ${\bar{p}}_{1}-{\bar{p}}_{2}$ = 0; $H_{1}$: ${\bar{p}}_{1}-{\bar{p}}_{2}\neq 0$.

**Table 4:** Percentage distribution of respondents with five distributive preferences (N = 500).

Preference Category	*t* = 1 Year	*t* = 2 Years	*t* = 5 Years	*t* = 20 Years	*t* = 50 Years
Concentration	7.2%	24.0%	37.0%	78.0%	74.6%
Diffusion	89.0%	63.2%	33.6%	4.0%	17.4%
Maximizing	3.4%	9.6%	21.2%	14.4%	4.0%
Extreme egalitarianism	0.2%	1.2%	6.8%	2.0%	1.6%
Extreme inequality seeking	0.2%	2.0%	1.4%	1.6%	2.4%
Total	100%	100%	100%	100%	100%

**Table 5:** PTO results—the social value of different health benefits (N = 432).

Health Gain (*t*) in Program A	Number of People (*P*) in Program A	Health Gain (*T*) in Program B	Indifference Number of People in Program B (Mean [SD])			
			Version 1 ($\bar{p_{1}}$) ^1^	Version 2 ($\bar{p_{2}}$) ^1^	*t*-Test (*p* Value) ^2^	Total ($\bar{p}$)
1	100	10	38.5 [1.3]	37.8 [1.4]	0.3736	38.0 [20.2] *
2	50	10	18.4 [0.7]	17.3 [0.7]	0.155	17.4 [10.8] *
5	20	10	9.5 [0.3]	10.2 [0.3]	0.9475	9.4 [3.9] *
20	5	10	32.5 [2.2]	35.1 [2.2]	0.7900	32.2 [27.7] *
50	2	10	38.7 [2.6]	41.1 [2.8]	0.7390	37.7 [35.6] *

Note: SD: standard deviation. ^1^ In questionnaire version 1, the five PTO scenarios appear in order of *t* from smallest to largest. In questionnaire version 2, the five PTO scenarios appear in order of *t* from largest to smallest. ^2^ $H_{0}$: $\bar{p_{1}}-\bar{p_{2}}$ = 0; $H_{1}$: $\bar{p_{1}}-\bar{p_{2}}\neq 0$. * Significant at the 5% level; $H_{0}$: $\bar{p}$ = 10; $H_{1}$: $\bar{p}\neq 10$.

**Table 6:** Utility function regression results.

ln u(*t*)	Coef.	SE	*p*	95%CI
*t*	0.035	0.002	<0.000	(0.030, 0.039)
ln *t*	0.646	0.030	<0.000	(0.587, 0.705)
constant	0.735	0.032	<0.000	(0.672, 0.798)
R^2^(adjusted)	0.771			

Note: *t*: health gains that each patient received in program A; u(*t*): the social value of *t*; *p*: *p*-value in statistical test; SE: standard error; CI: confidence interval.

## Data Availability

The datasets used and/or analyzed during the current study are available from the corresponding author upon reasonable request.

## Supplementary Materials

The following supporting information can be downloaded at: https://www.mdpi.com/article/10.3390/healthcare13111309/s1, Supplementary Material S1: Graphical representation of the iterative process in the person trade-off task; Supplementary Material S2: Quota standards; Supplementary Material S3: Pilot survey; Supplementary Material S4: The questionnaire; Supplementary Material S5: Respondent characteristics (N = 500); Supplementary Material S6: Distribution of the number of respondents for the indifference number of beneficiaries for program B (*p*); Supplementary Material S7: Test–retest results for five PTO tasks; Supplementary Material S8: Subgroup analysis results.

## Abbreviations

The following abbreviations are used in this manuscript.

QALYs	Quality-adjusted life years
CEA	Cost-effectiveness analysis
SWF	Social welfare function
HRSWF	Health-related social welfare function
LE	Life expectancy
UK	United Kingdom
PTO	Person trade-off
