# Supplementary material for: Eliciting Distributive Preferences in Health Care Resource Allocation: A Person Trade-Off Study
Source: Healthcare (Basel). 2025 May 30;13(11):1309. doi: 10.3390/healthcare13111309 (PMC12154901; doi:10.3390/healthcare13111309)
Supplement: Supplementary file 1 [file healthcare-13-01309-s001.zip › healthcare-3603811-supplementary.pdf]

Eliciting distributive preferences in health care resource allocation: A person trade-off study

Supplementary Materials

Contents

Supplementary Material S1: Graphical representation of the iterative process in the person trade-off task 2
Supplementary Material S2: Quota standards. 4
Supplementary Material S3: Pilot survey 5
Supplementary Material S4: The questionnaire 7
Supplementary Material S5: Respondent characteristics（N=500） 9
Supplementary Material S6: Distribution of the number of respondents for the indifference number of beneficiaries for program B (p). 11
Supplementary Material S7: Test–retest results for five PTO tasks. 14
Supplementary Material S8: Subgroup analysis results 16

Supplementary Material S1: Graphical representation of the iterative process in the person trade-off task

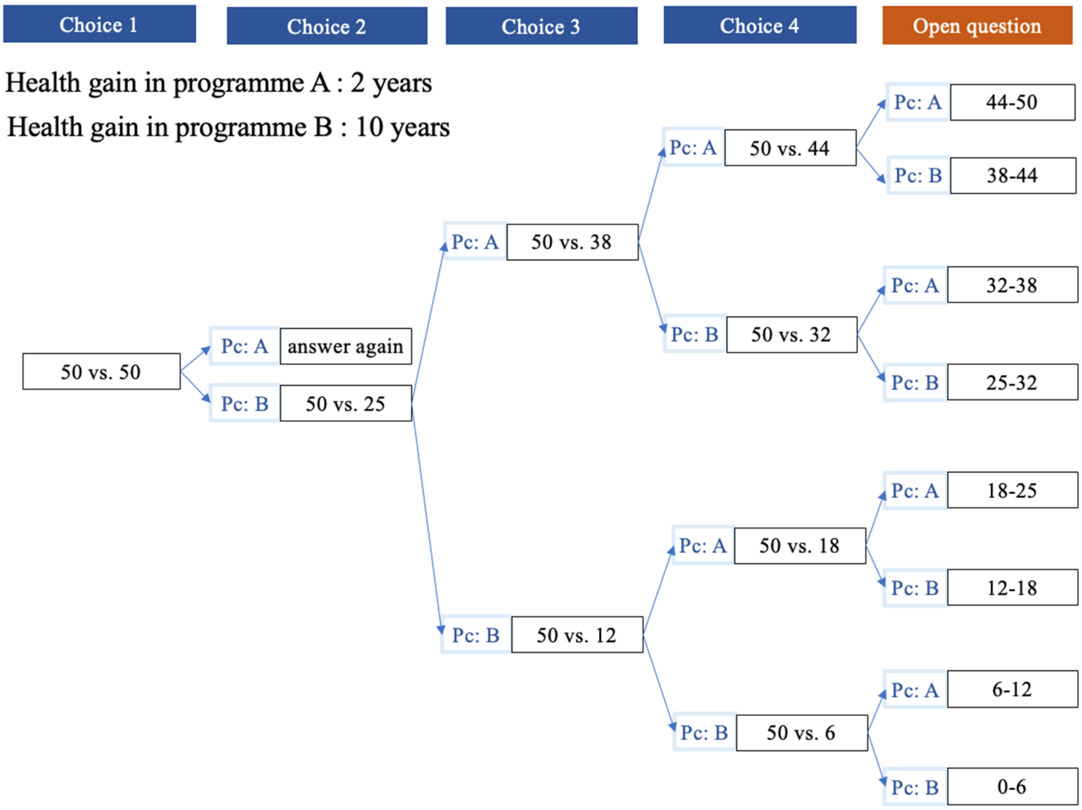

Figure S1 Graphical representation of the iterative process in PTO task when t= 2 years

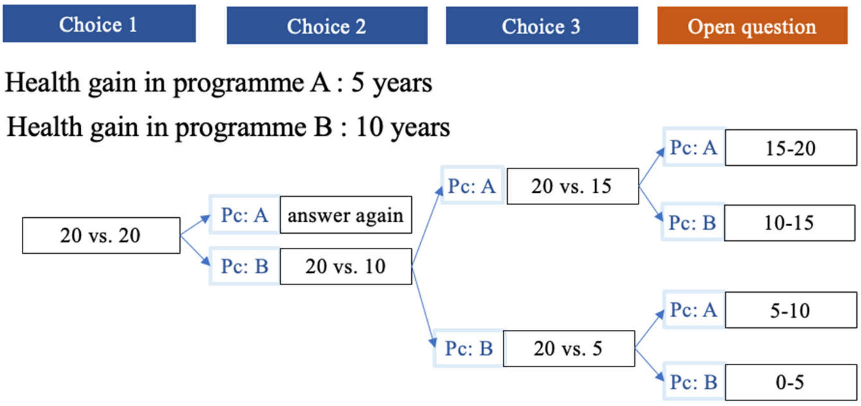

Figure S2 Graphical representation of the iterative process in PTO task when t= 5 years

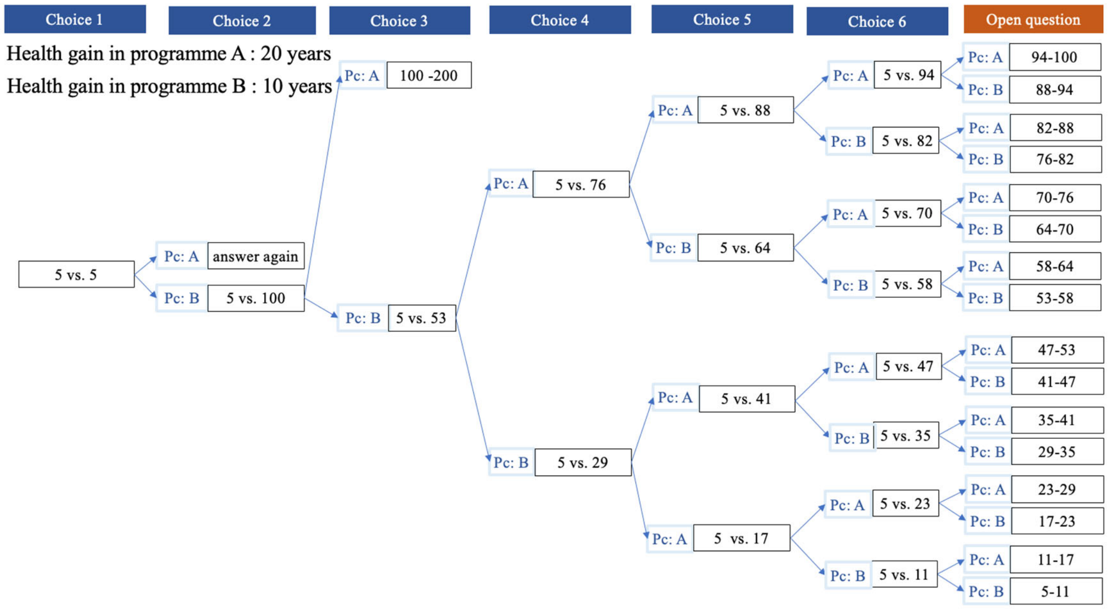

Figure S3 Graphical representation of the iterative process in PTO task when t= 20 years

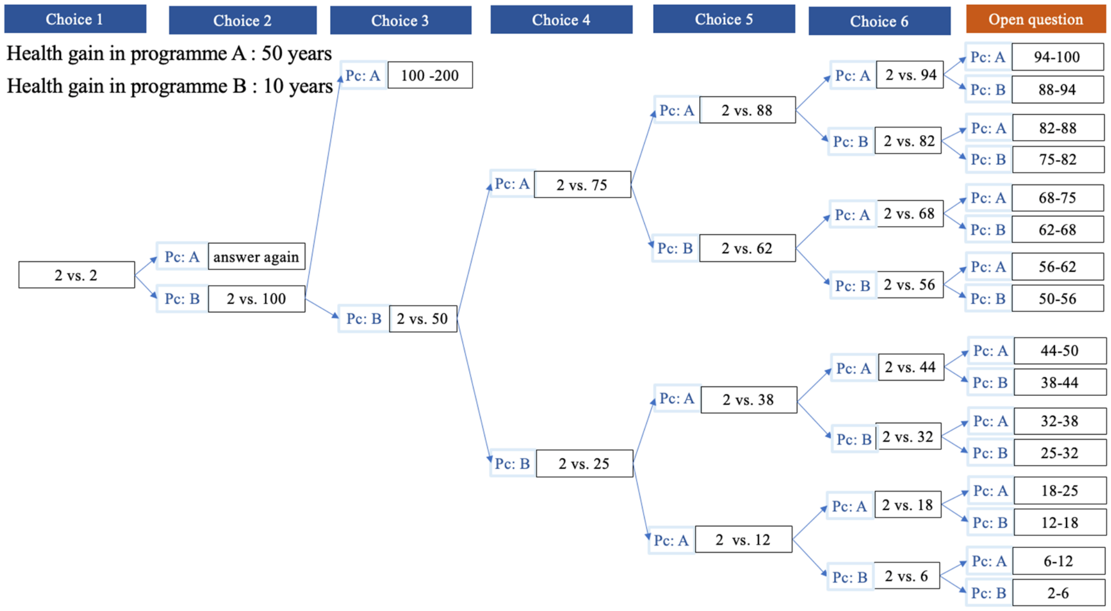

Figure S4 Graphical representation of the iterative process in PTO task when t= 50 years

Supplementary Material S2: Quota standards

Table S1 The quota standards in the main PTO online survey

**Characteristics**	Percentage	Northern China	North-East	Eastern China	Central China	Southern China	South-West	North-West	Total
		13.19%	7.67%	32.99%	17.41%	4.69%	15.98%	8.06%	100.00%
**Sex**									
Male	50.62%	33	19	83	44	12	40	20	253
Female	49.38%	33	19	81	43	12	39	20	247
**Age(years)**									
18-29	17.55%	12	7	29	15	4	14	7	88
30-39	20.07%	13	8	33	17	5	16	8	100
40-49	18.63%	12	7	31	16	4	15	8	93
50-59	20.01%	13	8	33	17	5	16	8	100
⩾60	23.74%	16	9	39	21	6	19	10	119
**Educational qualifications**									
Primary and below	25.03%	17	10	41	22	6	20	10	125
Junior High School	38.71%	26	15	64	34	9	31	16	194
High school (including secondary school)	17.03%	11	7	28	15	4	14	7	85
University (including college) and above	19.23%	13	7	32	17	5	15	8	96
**Place of residence type**									
Urban	63.89%	42	25	105	56	15	51	26	319
Rural	36.11%	24	14	60	31	8	29	15	181
**Total**		**66**	**38**	**165**	**87**	**23**	**80**	**40**	**500**

Supplementary Material S3: Pilot survey

Prior to our main survey， a face-to face pilot survey were run to test the comprehensiveness of the questionnaire and the face validity of the PTO tasks. A sample of Chinese adults (N=15) from three provinces in China (Jiangsu, Hebei and Gansu) was obtained through convenience sampling, most of whom were family and friends of the three researchers in our study. We also screened the sample characteristics of the pilot study to make it as representative of the population as possible. The characteristics of the sample are shown in the table below. Each respondent who participated in the pilot study was paid RMB 30. And the pilot study used a paper version of the questionnaire.

The results of pilot study shown that most respondents demonstrated high levels of understanding and completed the entire questionnaire. A small number of respondents noted that they were not able to understand the last open question immediately, thinking that it was still a choice question, so a detailed explanation of this question was added in the final version of the questionnaire. Further, to prevent answers from going outside the established range, the open questions were replaced with drop-down boxes.

Table S2 Characteristics of the sample in pilot study

Characteristics	Number of samples
**Sex**	
Male	8
Female	7
**Age(years)**	
18-29	3
30-39	3
40-49	3
50-59	3
≥60	3
**Educational qualifications**	
Primary and below	3
Junior High School	3
High school (including secondary school)	3
University (including college) and above	6
**Place of residence type**	
Urban	10
Rural	5
**Place of residence**	

Baoding, Hebei Province	5
Suzhou, Jiangsu Province	5
Linxia Hui Autonomous Prefecture, Gansu Province	5

Supplementary Material S4: The questionnaire

Taking health gains in program A is 1 year (t=1) as an example.

Now please consider the following scenarios:

(1) Suppose you currently need to allocate a health care budget to fund **one of the following two treatment programs**, both of which are used to treat some **20-year-old patients**.

(2) The patients treated with program A will **live additional 1 year**, and the patients treated with program B will live an additional **10** years, and the patients will be able to **live in full health** during the additional survival period.

(3) **The number of patients treated by program A is** 100, and the number of patients treated by program B will keep changing.

1. The number of patients that can be treated by both program A and program B is 100.

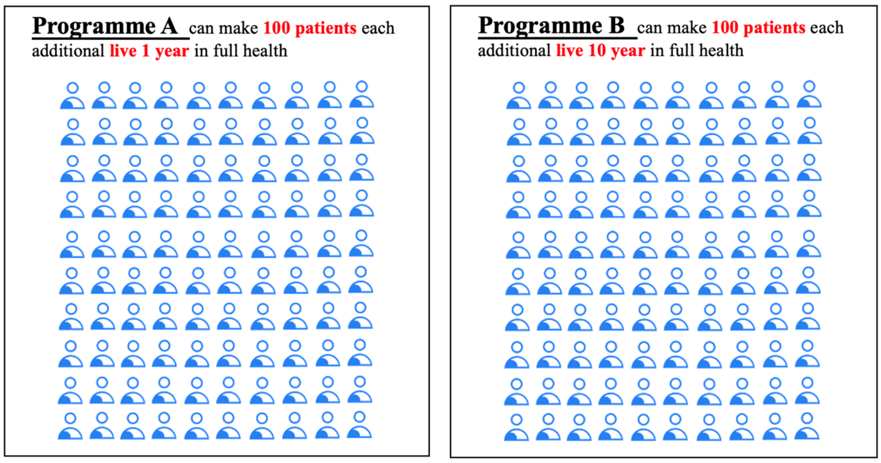

Which of the two programs above would you like to fund? Tick the options according to your choice. (In the answer to the following question, if you want to change your answer to the previous question, you can click the "Go Back" button at the bottom of the screen)

☐ Program A【Exit questionnaire】 ☐ Program B【Skip to question 2】

Go Back

2. Now, Program A is the same, but the number of patients that can be treated with Program B is down to 50.

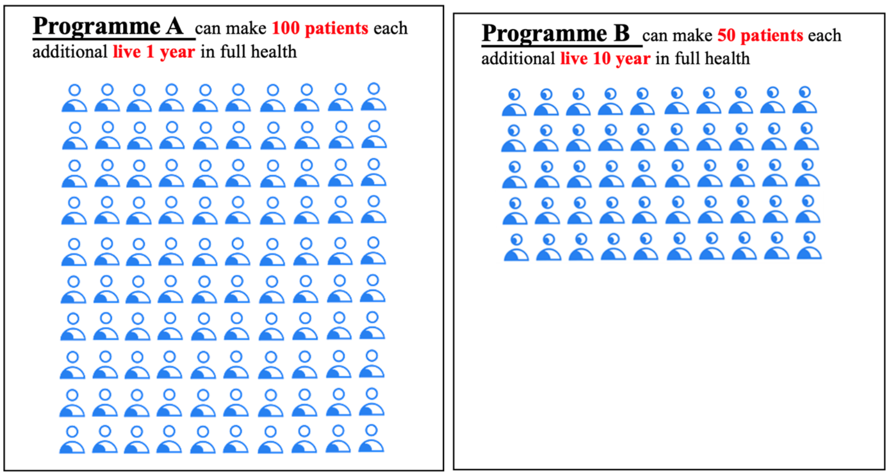

Which of the two programs above would you like to fund? Tick the options according to your choice.

☐ Program A【Skip to question 3】 ☐ Program B【Skip to question 4】

Go Back

3-16. ……

【Using a dichotomy to continuously increase or decrease the number of people treated with program B, until the respondents' indifference points between the two programs was locked within a range of about 5 people】

17. Based on your answers to the above questions, we can tell that you think:

When program A can treat 100 patients and program B can treat__**(upper limit of the range)**__patients, you think program **B is better**; and when program B can treat__**(lower limit of the range)**__patients, you think program A is better.

Then, please answer the question, when the number of patients that can be treated by program B is _________【fill in the blank】 people in the range _____*____, you think that there is no difference between program A and program B.

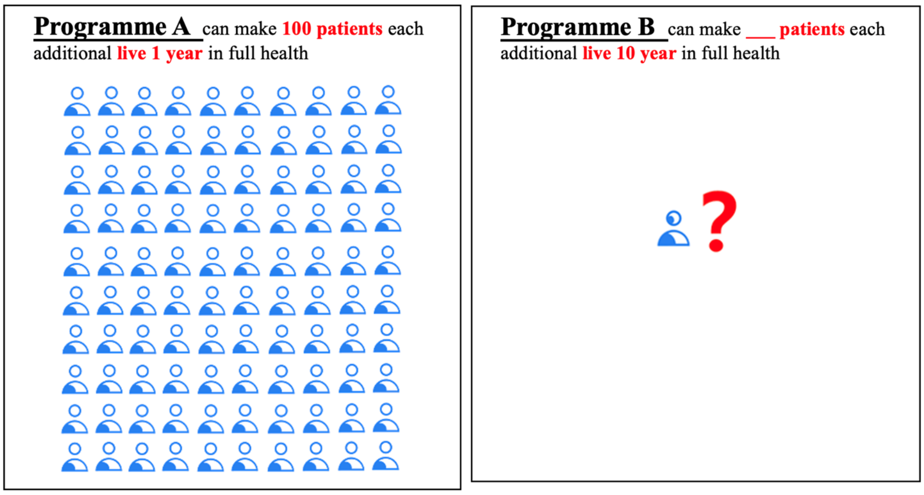

Go Back

【A drop-down box with numbers in the following range appears in the * position】

a 94-100 b 88-94 c 82-88 d 75-82 e 68-75 f 62-68 g 56-62 h 50-56

i 44-50 j 38-44 k 32-38 l 25-32 m 18-25 n 12-18 o 6-12 p 0-6

(The range of the number of indifference points is based on the results of previous choices)

Supplementary Material S5: Respondent characteristics（N=500）

Table S3 Characteristics of the Respondents in the main survey (N=500)

**Characteristics**	**n (%)**
**Age(years), [Mean (SD)]**	44.73(14.33)
18-29	88(17.6%)
30-39	100(20.0%)
40-49	93(18.6%)
50-59	100(20.0%)
⩾60	119(23.8%)
**Sex**	
Male	251(50.2%)
Female	249(49.8%)
**Educational qualifications**	
Primary and below	124(24.8%)
Junior High School	194(38.8%)
High school (including secondary school)	86(17.2%)
University (including college) and above	96(19.2%)
**Marital status**	
Single	60(12.0%)
Married	436(87.2%)
Divorced	2(0.4%)
Widowed	2(0.4%)
**Employment status**	
Employed (full-time or part-time)	419(83.8%)
Retired	52(10.4%)
In full-time education	22(4.4%)
Unemployed	7(1.4%)
**Personal monthly income**	
<¥2,000	5(1.0%)
¥2,000-5,000	274(54.8%)
¥5,000-10,000	163(32.6%)
>¥10,000	58(11.6%)
**Place of Residence type**	
Urban	319(63.8%)
Rural	181(36.2%)
**Medical insurance type**	
UEBMI	359(71.8%)
URRBMI	154(30.8%)
Commercial medical insurance	153(30.6%)
None	15(3.0%)

**Self-reported general health**	
Very good	73(14.6%)
Good	242(48.4%)
Fair	170(34.0%)
Bad	15(3.0%)
Very bad	0(0.0%)
**Area of residence in China**	
Northern China	66(13.2%)
North-East	38(7.6%)
Eastern China	165(33.0%)
Central China	87(17.4%)
Southern China	23(4.6%)
South-West	80(16.0%)
North-West	41(8.2%)

UEBMI：Urban Employee Basic Medical Insurance；URRBMI：Urban and Rural Residents Basic Medical Insurance

Supplementary Material S6: Distribution of the number of respondents for the indifference number of beneficiaries for program B (p)

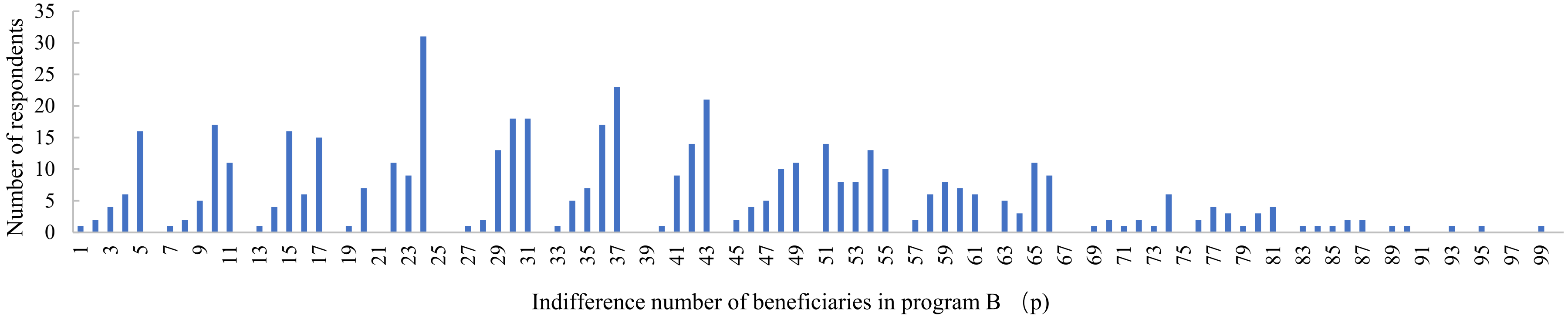

Figure S5 Distribution of the number of respondents for p when t=1 year

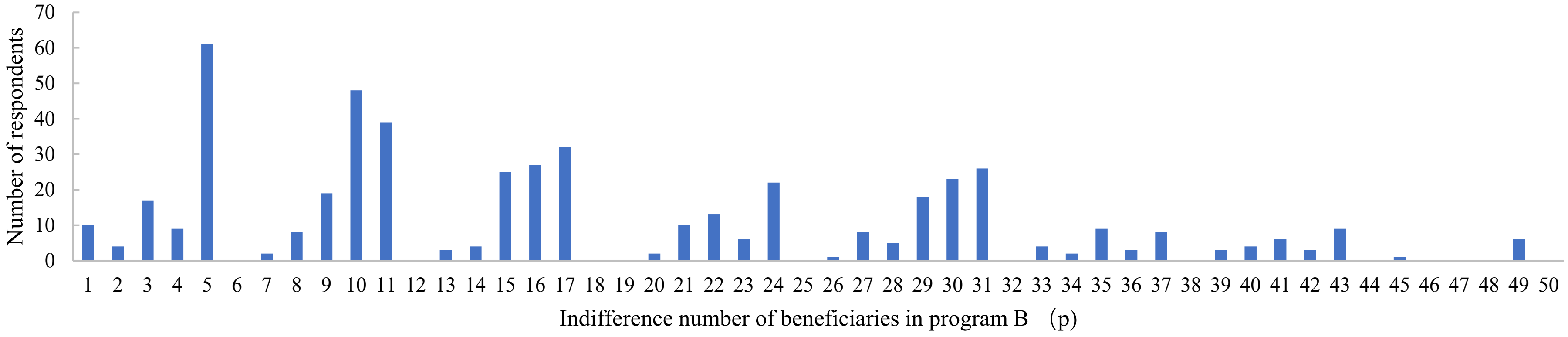

Figure S6 Distribution of the number of respondents for p when t=2 years

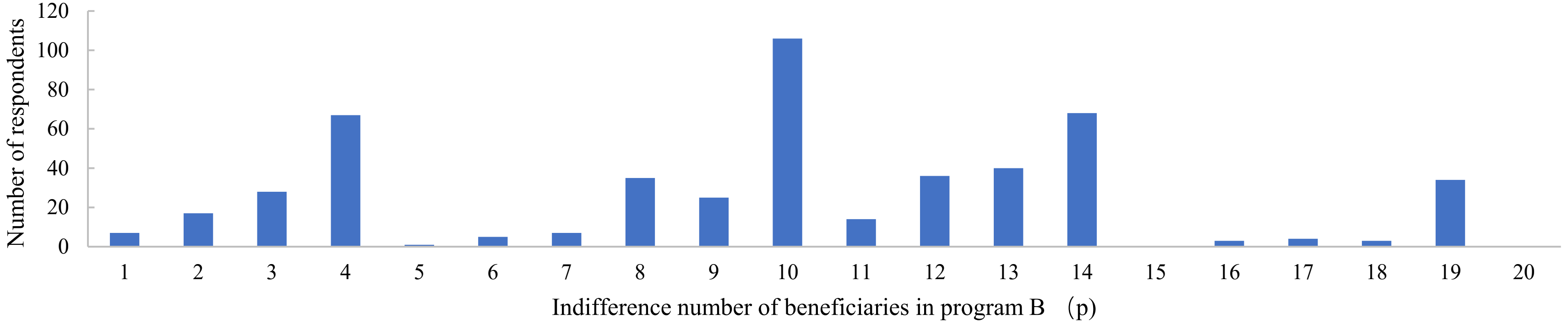

Figure S7 Distribution of the number of respondents for p when t=5 years

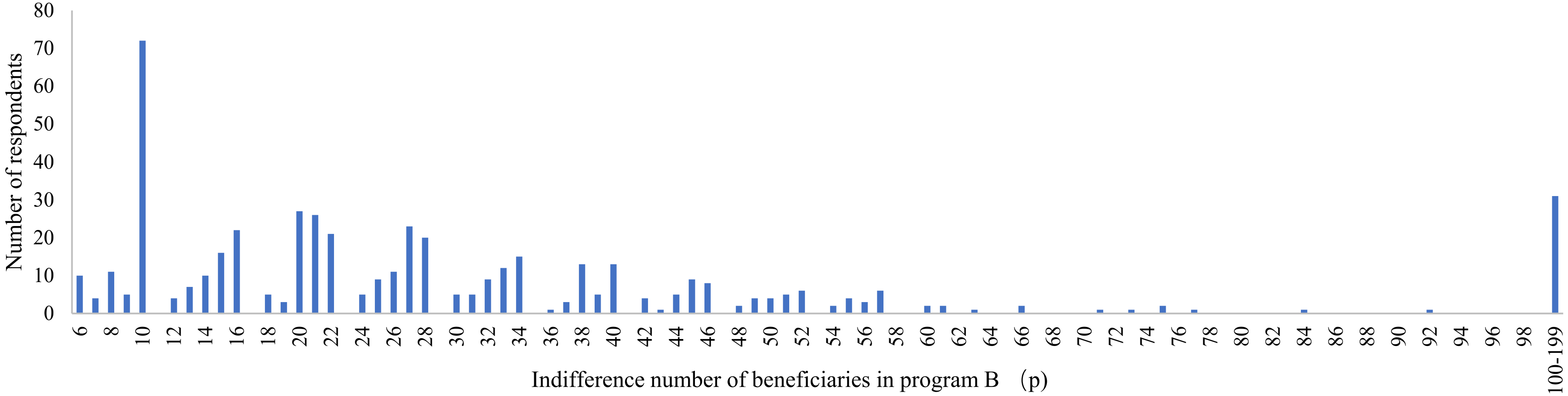

Figure S8 Distribution of the number of respondents for p when t=20 years

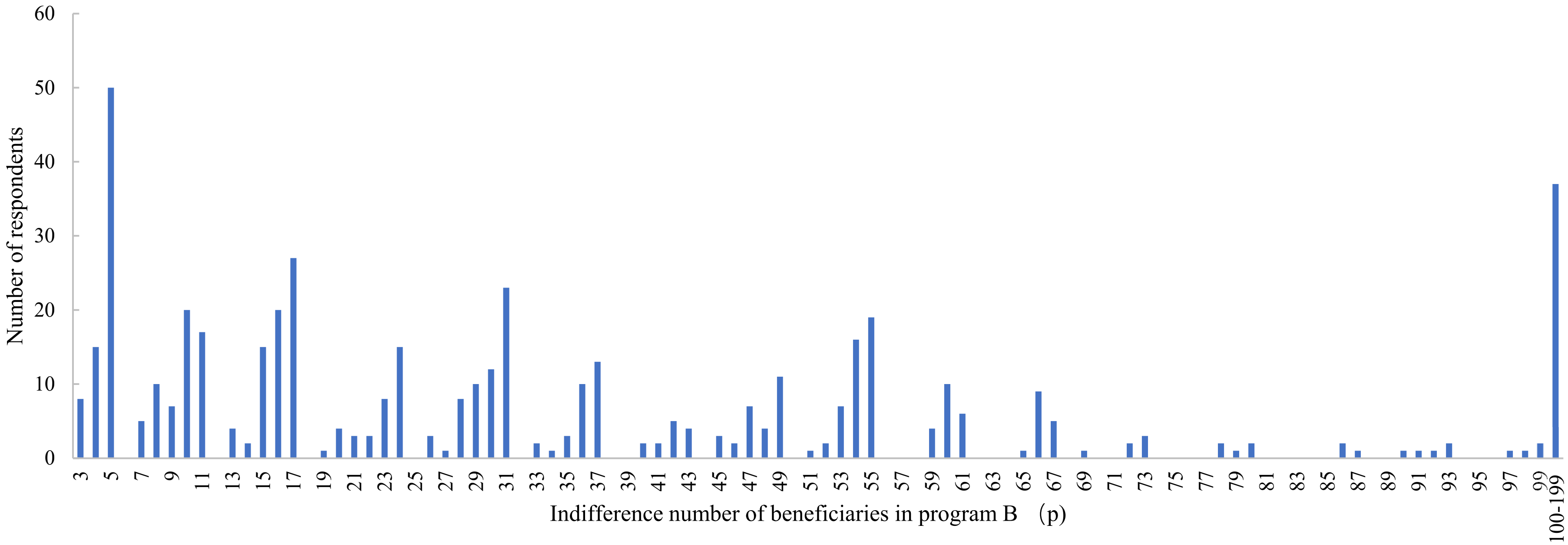

Figure S9 Distribution of the number of respondents for p when t=50 years

Supplementary Material S7: Test–retest results for five PTO tasks

Table S4 The result of intra-class correlation coefficient (ICC)

Health benefit in program A (t) (years in full health)	Indifference number of people in program B (p) (Mean [SE])		ICC (95%CI)
	First round	Second round	
1	40.03(1.37)	41.36(1.44) *	0.728(0.662,0.782)
2	18.40(0.76)	18.02(0.75) *	0.875(0.842,0.902)
5	9.95(0.28)	10.50(0.31) *	0.652(0.573,0.719)
20	32.93(2.07)	32.78(2.26) *	0.387(0.275,0.489)
50	36.81(2.18)	35.79(2.65) *	0.422(0.313,0.521)

* Significative at 5% level; H_0: Difference of means of p in two rounds survey=0; H_1:Difference of means of p in two rounds survey≠0；ICC：intra-class correlation coefficient；SE：standard error

ICC values of between 0 and 0.5 are considered to represent poor reliability, between 0.5 and 0.75 represent moderate reliability, between 0.75 and 0.9 good represent reliability, and >0.9 represent excellent reliability.

The results of individual-level data analysis shown that, most of the respondents could repeat their choice entirely and their answers were the same between the two round surveys.

(a) t= 1 year

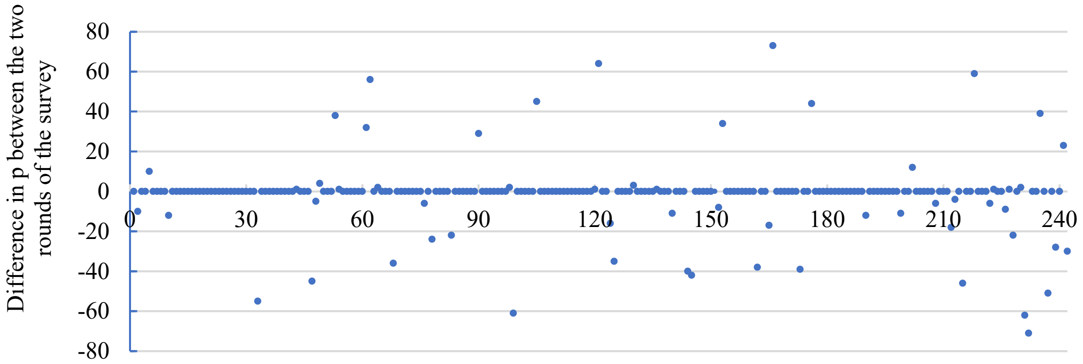

(b) t= 2 years

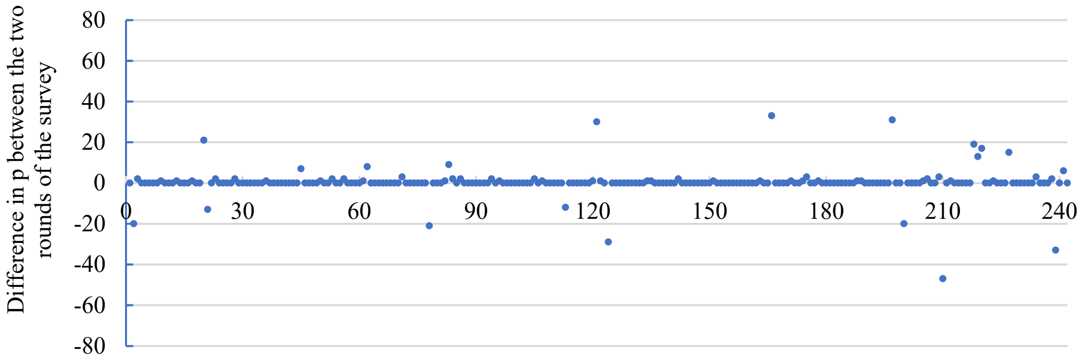

(c) t= 5 years

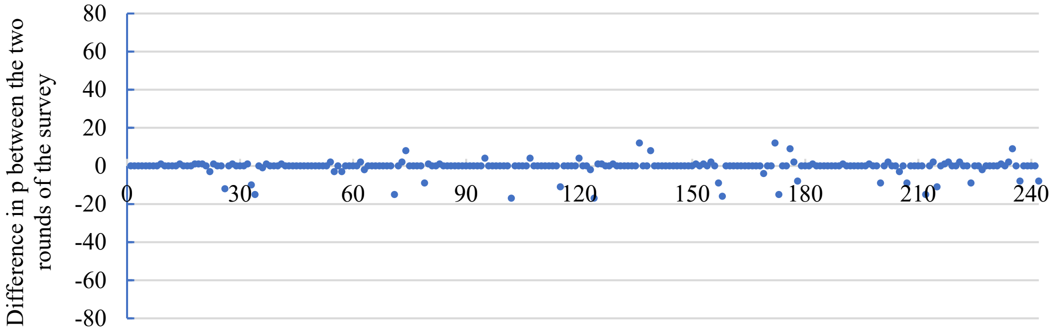

(d) t= 20 years

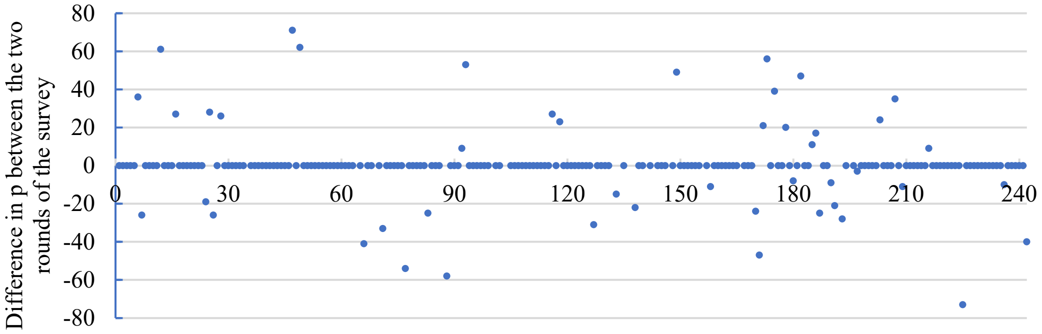

(e) t= 50 years

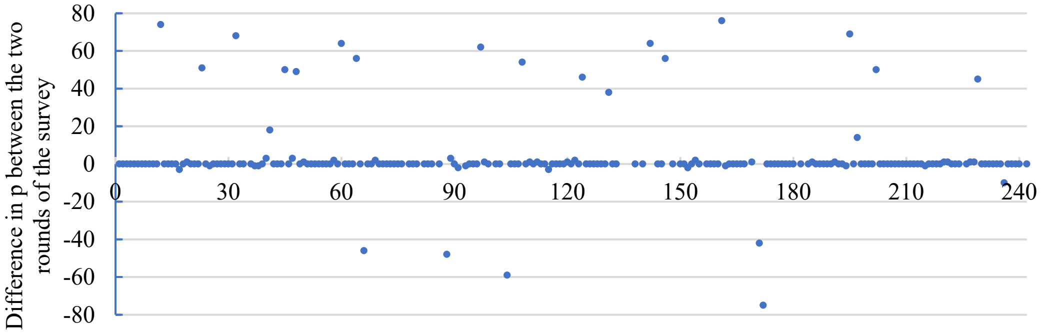

Figure S10 The difference between the indifference number of people who benefited from program B(p) in the first round and the second-round survey (N=242)

Supplementary Material S8: Subgroup analysis results

(1) Income level

Table S5 Subgroup analysis for different personal monthly income level respondents

Income level (CN¥)	Sample size	Sample size after exclusion [a]	Indifference number of people in program B ($\bar{p}$)				
			t=1	t=2	t=5	t=20	t=50
All	**500**	**432**	**38**	**17.4**	**9.4**	**32.2**	**37.7**
< 5000	279	239	41.2*	19.1*	9.8	29.7	34.3
5000- 10000	163	144	34.5*	14.3***	8.2***	37.4	46.8*
> 10000	58	49	33	17.8	10.6**	29.3	27.2*

A The non-trade-off samples were excluded; "t" refers to the health gains that each patient received in program A

*p<0.1, **p<0.05, ***P<0.01

(a) The curve of u(t)

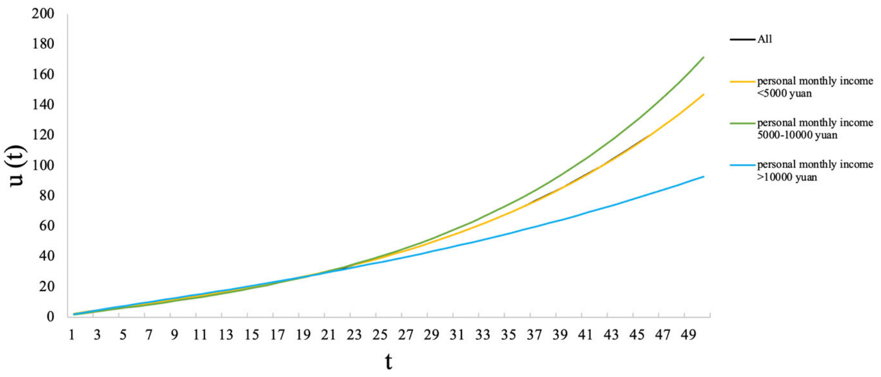

(b) The curve of u''(t)

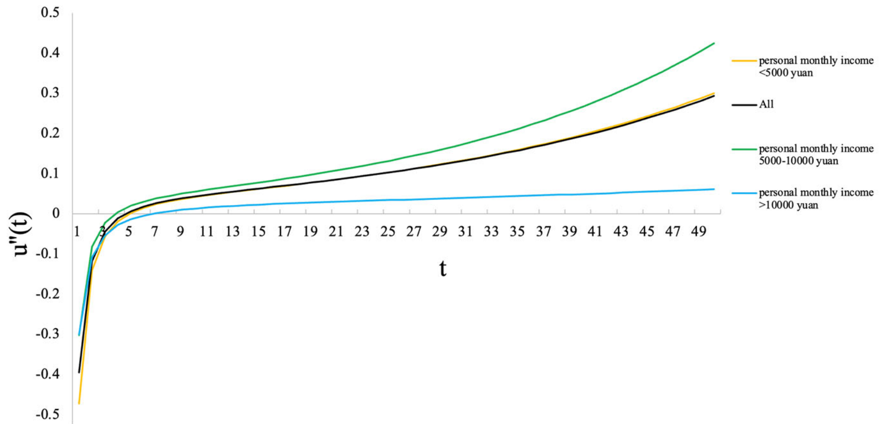

Figure S11 The curve of u (t) and u''(t) for different personal monthly income level respondents

Table S6 The size of the threshold of health gains, when the distributive preferences change

Personal monthly income level	Threshold (years)
All	4.6
<5000	4.9
5000-10000	4.9

>10000	6.7

(2) Age

Table S7 Subgroup analysis for different age respondents

Age	Sample size	Sample size after exclusion [a]	Indifference number of people in program B ($\bar{p}$)				
			t=1	t=2	t=5	t=20	t=50
All	**500**	**432**	**38**	**17.4**	**9.4**	**32.2**	**37.7**
18-29	88	73	39.4	17.8	9.7	30.5	35.8
30-39	100	83	34.4	17.2	9.8	28.1	32.1
40-49	93	83	40	19.1	9	33.5	34.9
50-59	100	87	41	18	8.8	42.5	47.7
≥60	119	106	36	15.5	9.6	27.1**	37.2

A The non-trade-off samples were excluded; "t" refers to the health gains that each patient received in program A

*p<0.1, **p<0.05, ***P<0.01

(a) The curve of u(t)

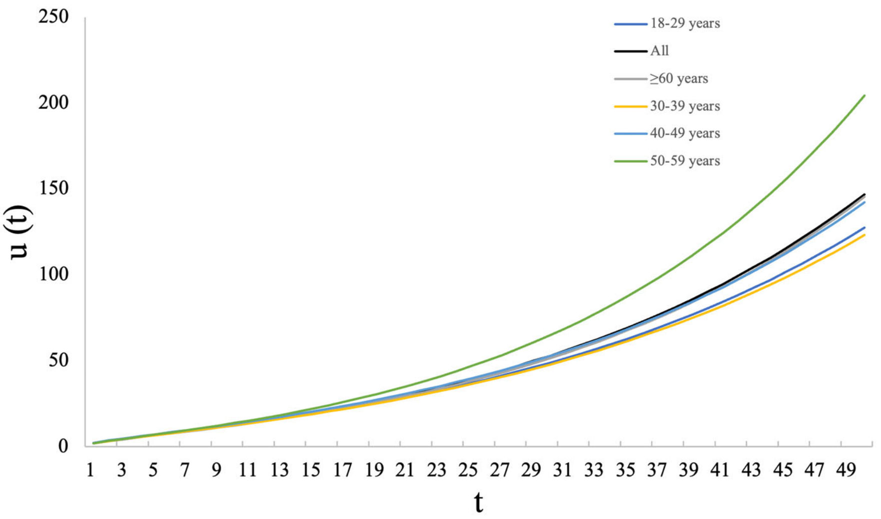

(b) The curve of u''(t)

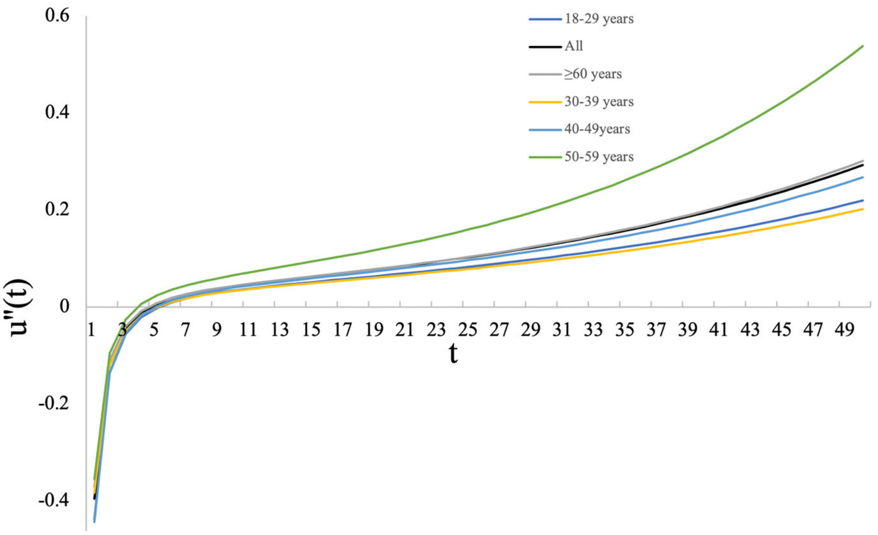

Figure S12 The curve of u(t) and u''(t) for different age respondents

Table S8 The size of the threshold of health gains, when the distributive preferences change

Personal monthly income level	Threshold (years)
All	4.6
18-29	5.2
30-39	5.1
40-49	4.9
50-59	3.8
≥60	4.5

(3) Self-reported health status

Table S9 Subgroup analysis for different self-reported health status

Age	Sample size	Sample size after exclusion [a]	Indifference number of people in program B ($\bar{p}$)				
			t=1	t=2	t=5	t=20	t=50
All	**500**	**432**	**38**	**17.4**	**9.4**	**32.2**	**37.7**
Very good	73	60	39.9	18.3	10.3	34	38.1
Good	242	209	41.6**	19.1*	9.8	33	37.8
General	170	151	33.5**	15**	8.6**	29.3	36.3
Poor	15	12	24.2*	11.8	7.9	47.1	50

A The non-trade-off samples were excluded; "t" refers to the health gains that each patient received in program A

*p<0.1, **p<0.05, ***P<0.01

(a) The curve of u(t)

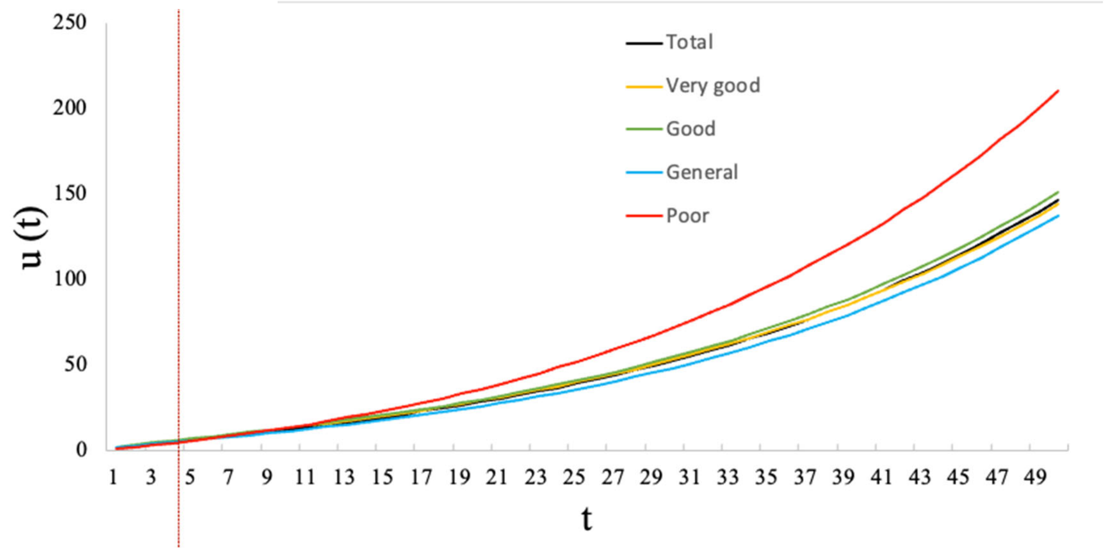

(b) The curve of u’’(t)

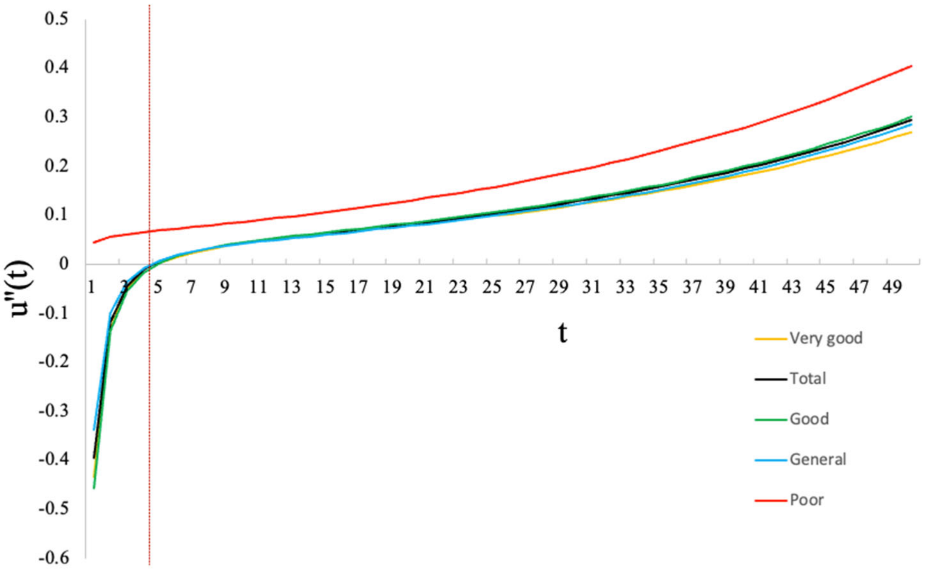

Figure S13 The curve of u(t) and u’’(t) for different self-reported health status

Table S10 The size of the threshold of health gains, when the distributive preferences change

Personal monthly income level	Threshold (years)
All	4.6
Very good	4.9
Good	4.9
General	4.4
Poor	NA

(4) Education level

Table S11 Subgroup analysis for different education level

Age	Sample size	Sample size after exclusion [a]	Indifference number of people in program B ($\bar{p}$)				
			t=1	t=2	t=5	t=20	t=50
All	**500**	**432**	**38**	**17.4**	**9.4**	**32.2**	**37.7**
Primary and below	124	111	43.7**	19.3	9.6	28.8	34.2
Junior High School	194	169	35	15.6	9.4	38.2	44.7
High school (including secondary school)	86	70	42.1	19.5	9.4	33	36.7
University (including college) and above	96	82	33.2*	15.9	9.2	31.7	36.8

a The non-trade-off samples were excluded; “t” refers to the health gains that each patient received in program A

*p<0.1,**p<0.05,***P<0.01

(a) The curve of u(t)

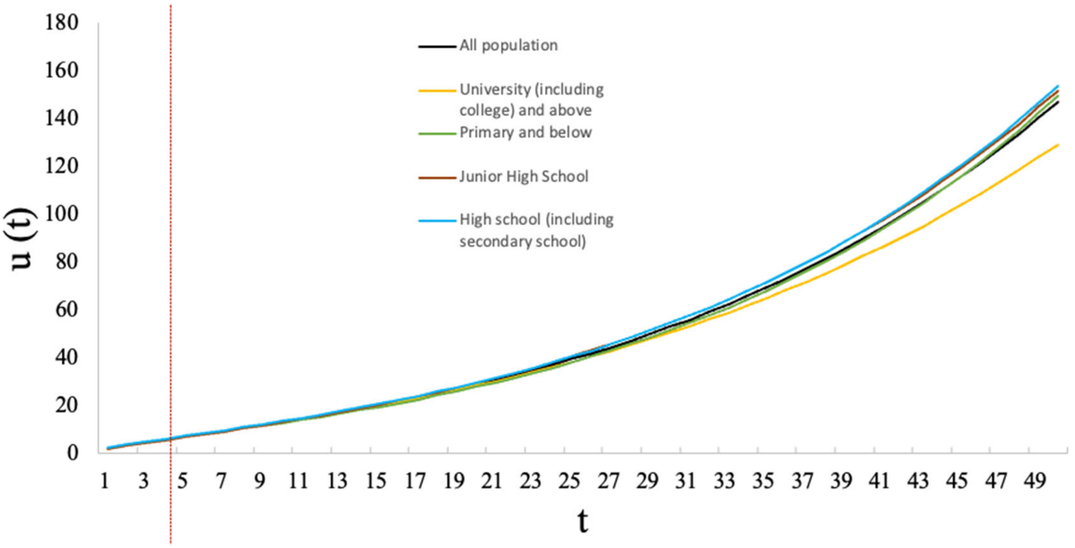

(b) The curve of u''(t)

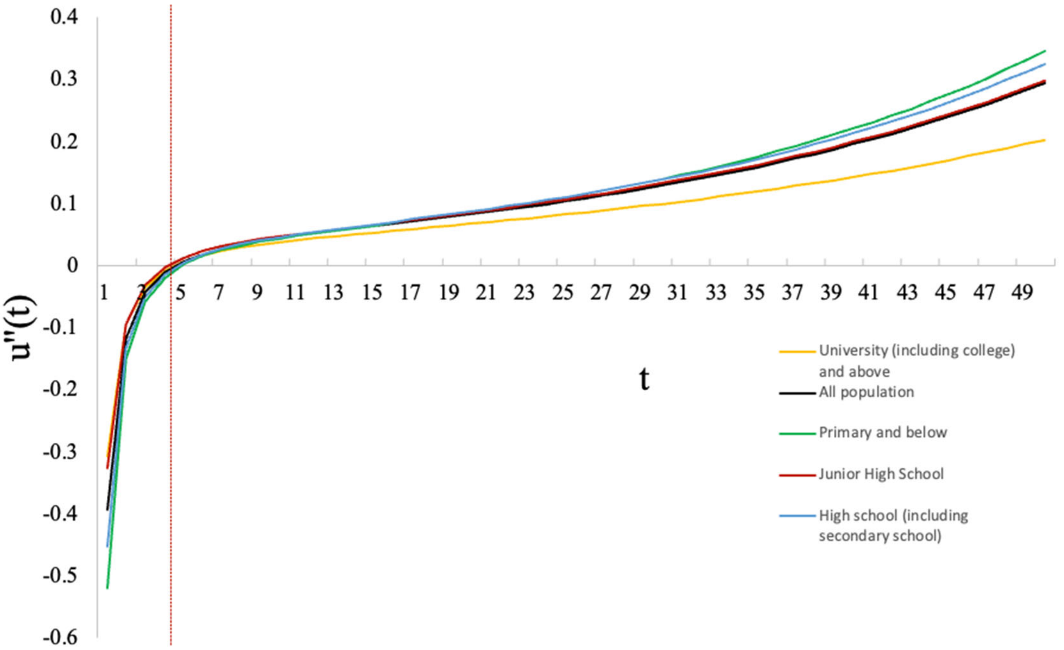

Figure S14 The curve of u(t) and u''(t) for different education level

Table S12 The size of the threshold of health gains, when the distributive preferences change

Personal monthly income level	Threshold (years)
All	4.6
Primary and below	4.9
Junior High School	4.2
High school (including secondary school)	4.7
University (including college) and above	4.6
